# Antibacterial and anti-virulence effects of furazolidone on *Trueperella pyogenes* and *Pseudomonas aeruginosa*

**DOI:** 10.1186/s12917-022-03216-5

**Published:** 2022-03-24

**Authors:** Qin Chen, Kelei Zhao, Heyue Li, Kanghua Liu, Jing Li, Yiwen Chu, Balakrishnan Prithiviraj, Bisong Yue, Xiuyue Zhang

**Affiliations:** 1 Key Laboratory of Bio-resources and Eco-environment, Ministry of Education, College of Life Sciences, Sichuan University, No. 24, South Section 1, Yihuan Road, Chengdu, 610064 Sichuan PR China; 2 Antibiotics Research and Re-evaluation Key Laboratory of Sichuan Province, School of Pharmacy, Chengdu University, No. 2025, Chengluo Avenue, Chengdu, 610106 Sichuan PR China; 3 Marine Bio-products Research Laboratory, Department of Plant, Food and Environmental Sciences, Dalhousie University, Truro, NS Canada

**Keywords:** Forest musk deer, *Trueperella pyogenes*, *Pseudomonas aeruginosa*, Quorum-sensing, Furazolidone, Anti-virulence

## Abstract

**Background:**

*Trueperella pyogenes* and *Pseudomonas aeruginosa* are two important bacterial pathogens closely relating to the occurrence and development of forest musk deer respiratory purulent disease. Although *T. pyogenes* is the causative agent of the disease, the subsequently invaded *P. aeruginosa* will predominate the infection by producing a substantial amount of quorum-sensing (QS)-controlled virulence factors, and co-infection of them usually creates serious difficulties for veterinary treatment. In order to find a potential compound that targets both *T. pyogenes* and *P. aeruginosa*, the antibacterial and anti-virulence capacities of 55 compounds, which have similar core structure to the signal molecules of *P. aeruginosa* QS system, were tested in this study by performing a series of in vitro screening experiments.

**Results:**

We identified that furazolidone could significantly reduce the cell densities of *T. pyogenes* in mono-culture or in the co-culture with *P. aeruginosa*. Although the growth of *P. aeruginosa* could also be moderately inhibited by furazolidone, the results of phenotypic identification and transcriptomic analysis further revealed that sub-inhibitory furazolidone had remarkable inhibitory effect on the biofilm production, motility, and QS system of *P. aeruginosa*. Moreover, furazolidone could efficiently protect *Caenorhabditis elegans* models from *P. aeruginosa* infection under both fast-killing and slow-killing conditions.

**Conclusions:**

This study reports the antibacterial and anti-virulence abilities of furazolidone on *T. pyogenes* and *P. aeruginosa*, and provides a promising strategy and molecular basis for the development of novel anti-infectious drugs to dealing with forest musk deer purulent disease, or other diseases caused by *T. pyogenes* and *P. aeruginosa* co-infection.

**Supplementary Information:**

The online version contains supplementary material available at 10.1186/s12917-022-03216-5.

## Background

Forest musk deer (*Moschus berezovskii*) is an important economic animal endemic in China, and has been categorized as first-class key species of wildlife protected by Chinese legislation in 2002. The musk secreted by male forest musk deer is a precious Chinese medicine and important raw material of high-grade spice. As a kind of solitary small ruminant, forest musk deer are timorous and fiddle-footed and easily hurt themselves while leaping. These casual injuries frequently lead to the occurrence of purulent disease, which normally manifests as suppurative lesions on the epidermis, uterus, and internal organs [1, 2]. Previous studies have revealed that purulent disease is one of the main reasons hindering the increase of forest musk deer population and causes more than 50% of the total deaths [3, 4]. In a farm with ≈ 400 forest musk deer in Sichuan Province (China), 8–10 individuals are raised in each fold about 200 m^2^. According to the statistical report of forest musk deer therapy, we find that among the 467 treatment records (including 100 death cases) in about 2 years, 165 of them (35.3%) were related to purulent disease, and 41 death cases were diagnosed as severe internal suppuration, especially in the lung. Compared to the body surface abscesses which can be easily observed and removed artificially, the internal suppurative lesions are usually fatal and difficult to be detected in time [1, 2, 5].

Our prior work has shown that *Trueperella pyogenes* and *Pseudomonas aeruginosa* are two main bacterial pathogens in the respiratory suppurative lesion of forest musk deer. *T. pyogenes* is considered as the primary pathogen, while *P. aeruginosa* become the dominant species in the lateral stage [1, 4, 6]. The detection rate of *T. pyogenes* in the samples from surface abscesses and internal suppurative lesions of forest musk deer was 100% (28/28). Other bacterial species, such as *P. aeruginosa* (21.4%), *Escherichia coli* (7.2%), *Bacillus cereus* (14.3%), and etc., could only be isolated from the internal suppurative lesions [2]. *T. pyogenes* is a Gram-positive, pleomorphic, non-spore forming, inactive, non-enveloped and facultative anaerobic bacterium belonging to the family *Actinomycetaceae*, and a resident bacterium in the skin and mucous membranes of animal respiratory tract, digestive tract, and genitourinary tract [7–10]. The biosynthesis of the key virulence factor pyolysin is depended on fermentation metabolism of *T. pyogenes*. This process can reduce the oxygen content in the infection site and thus benefits the growth of other anaerobic or facultative anaerobic bacterial species [10–12].

*P. aeruginosa* is a ubiquitous opportunistic Gram-negative bacterium that can infect a variety of host tissues and cause acute and chronic infections [13, 14]. It is well-recognized that quorum-sensing (QS) system plays an important role in the processes of bacterial invasion and cell-cell communications. The QS system of many Gram-negative bacterial species is activated by specific signal molecules (acyl-homoserine lactones, AHLs), and coordinates the production of diverse virulence factors [5, 15–17]. In *P. aeruginosa*, the QS system is composed of three hierarchically arranged regulatory networks. The *las*- and *rhl*-QS systems have complete signal molecule synthesis proteins (LasI/RhlI) and regulatory proteins (LasR/RhlR). The activation of *rhl* is largely depended on the *las* system. The *pqs* system only has the regulatory protein *pqsR*, and the activation of which requires *Pseudomonas* quinolone signal from other pathways co-regulated by *lasR* and *rhlR* [16].

In the past years, aminoglycosides and cephalosporins were the antibiotics routinely used to treat the infection diseases of forest musk deer. However, the increased frequency of treatment failure caused by bacterial antibiotic resistance has brought huge challenge and economic losses to forest musk deer breeding industry [1, 2]. It is considered that inhibiting the virulence of multi-resistant bacteria by targeting the QS system, rather than killing them, is a promising strategy for the development of novel anti-infectious drugs, namely QS inhibitors or anti-virulence drugs [18]. *P. aeruginosa* has robust innate resistance to a wide range of antibiotics, and is a model species in the screening of anti-infectious compounds because of its well-characterized QS system [13, 15, 17]. We have previously shown that the natural AHL signals of *P. aeruginosa* QS system could inhibit the growth and virulence of multi-resistant *T. pyogenes* [19]. *P. aeruginosa* has strong competitive advantage over *T. pyogenes* when any of the three QS regulators of *P. aeruginosa* was knocked out [20]. Therefore, we hypothesize that there might be a kind of compound that has the potential to simultaneously control *T. pyogenes* and *P. aeruginosa*, either by inhibiting the growth or virulence of them. In this study, we tested the antibacterial and anti-QS activities of 55 small molecule compounds with similar core structure (furan, benzofuran, or flavonoids) to the AHL signals of *P. aeruginosa* (Additional file 1 Supplementary Fig. S1), or to the QS inhibitors identified previously [18]. Finally, we identified that furazolidone had strong inhibitory activity on *T. pyogenes* and moderately inhibited the growth of *P. aeruginosa*. Moreover, sub-inhibitory furazolidone showed a strong inhibitory activity on the QS system of *P. aeruginosa*.

## Results

### Screening of compounds inhibit the growth of *T. pyogenes*

**Fig. 1:**
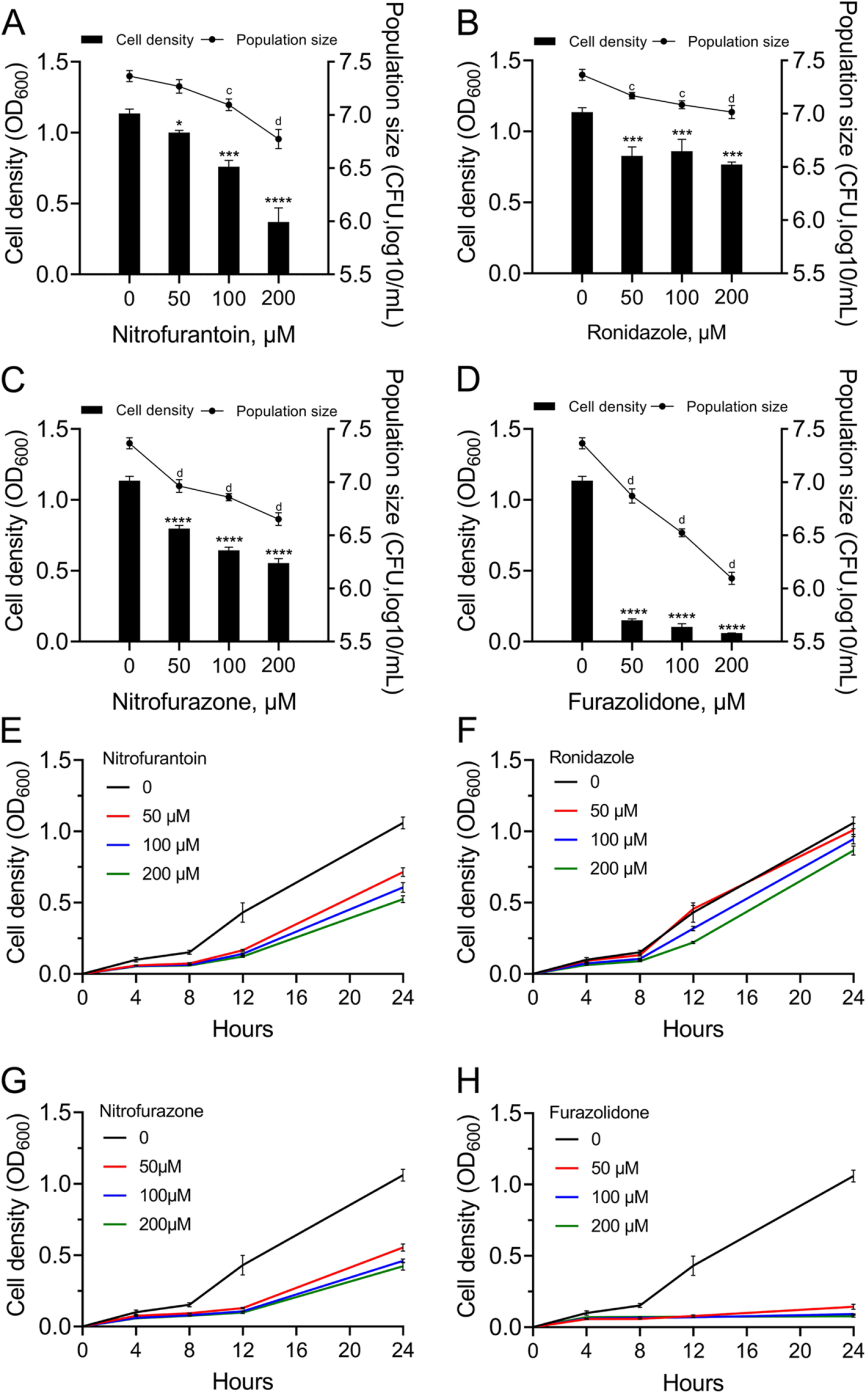
Dose- and time-dependent effect of different compounds on the growth of *Trueperella pyogenes* TP13. Equal amount of *T. pyogenes* TP13 was cultured at 37 °C for different time durations in brain heart infusion medium containing 5% fetal bovine serum (BHI-FBS) and different concentrations of (**A**, **E**) nitrofurantoin, (**B**, **F**) ronidazole, (**C**, **G**) nitrofurazone, or (**D**, **H**) furazolidone. Cell densities were determined by measuring the optical density (left Y-axis) at 600 nm (OD _600_) and colony forming units (CFUs) enumeration on BHI-FBS agar plates (right Y-axis). Data shown are the mean ± standard deviation (SD) of three independent experiments, compare to the untreated control. One-way ANOVA, * *p* < 0.05, *** *p* < 0.001, **** *p* < 0.0001. Small letters a and b above the symbols indicate *p* < 0.001 and *p* < 0.0001, respectively

For the 55 compounds with similar core structure to the AHL signals of *P. aeruginosa* QS system, we first tested the inhibitory effects of them on the growth of *T. pyogenes* TP13 in brain heart infusion medium containing 5% fetal bovine serum (BHI-FBS). The result showed that compared with the control, 9 compounds were found to have significant growth inhibition effect on *T. pyogenes* TP13 and 8 have significant growth enhancement effect (Additional file 1 Supplementary Table S1). Nitrofurantoin, nitrofurazone, ronidazole, and furazolidone, which could significantly inhibit *T. pyogenes* TP13 in a dose- and time-dependent manner (Fig. 1), were selected to treat co-cultured *T. pyogenes* and *P. aeruginosa*.

### Furazolidone inhibits the growth of *T. pyogenes* and *P. aeruginosa*

**Fig. 2:**
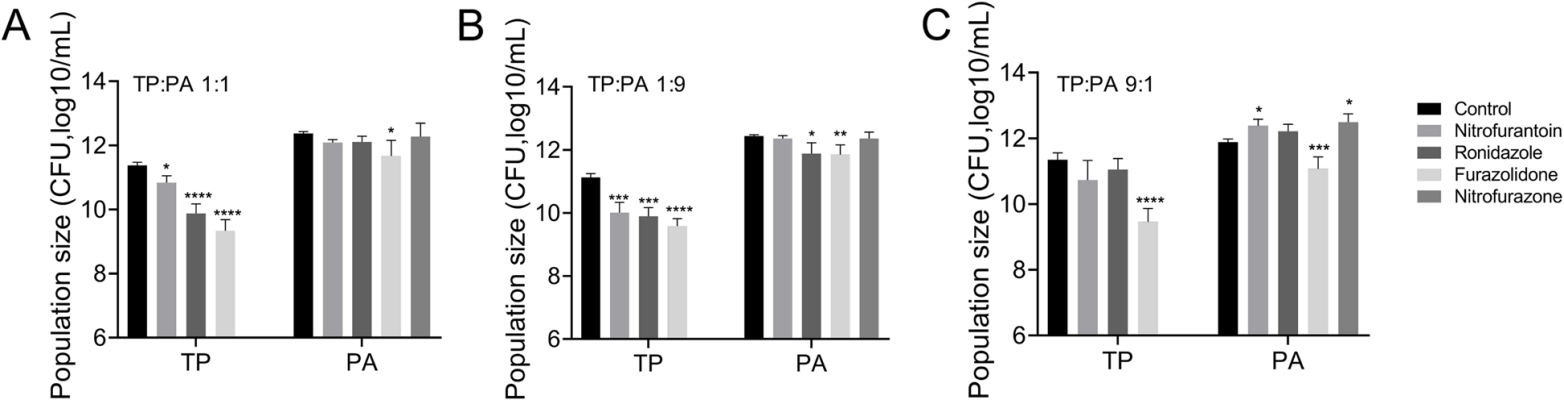
Effects of different compounds on the growth of *T. pyogenes* TP13 and *Pseudomonas aeruginosa* PAO1 under different co-culture conditions. *T. pyogenes* TP13 and *P. aeruginosa* PAO1 were mixed and co-cultured on BHI + 5% FBS plates containing 200 μM of nitrofurantoin, ronidazole, nitrofurazone, or furazolidone from the initial ratios of (**A**) 1:1, (**B**) 1:9, and (**C**) 9:1, and cultured overnight at 37 °C. Data shown are the mean ± SD of three independent experiments, compare to the untreated control. One-way ANOVA, * *p* < 0.05, ** *p* < 0.01, *** *p* < 0.001, **** *p* < 0.0001

*T. pyogenes* TP13 and *P. aeruginosa* PAO1 were well-mixed into different ratios (1:1, 1:9, and 9:1) and co-cultured on BHI-FBS agar supplemented with 200 μM of nitrofurantoin, ronidazole, furazolidone, or nitrofurazone. In agreement with our prior finding that *P. aeruginosa* had an innate growth advantage in the competition with *T. pyogenes* [19], *P. aeruginosa* would always be the dominant species under co-culture condition with *T. pyogenes*, irrespective of their initial ratios (Fig. 2). The addition of nitrofurantoin, ronidazole, furazolidone, or nitrofurazone significantly reduced the number of *T. pyogenes* TP13 cells in all the polymicrobial colonies compared to the control. Differently, compared to the co-culture of *T. pyogenes* TP13 and *P. aeruginosa* PAO1 started from the ratio of 9:1 on blank BHI-FBS plates, there were more *P. aeruginosa* PAO1 cells in the polymicrobial colony on the plates supplemented with nitrofurantoin or nitrofurazone. Notably, among the 4 tested compounds, only furazolidone could simultaneously inhibit *T. pyogenes* TP13 and *P. aeruginosa* PAO1 compared to the control, and the growth inhibition of furazolidone on *T. pyogenes* TP13 was stronger than that on *P. aeruginosa* PAO1 (Fig. 2).

### Furazolidone inhibits the QS-related phenotypes of *P. aeruginosa*

**Fig. 3:**
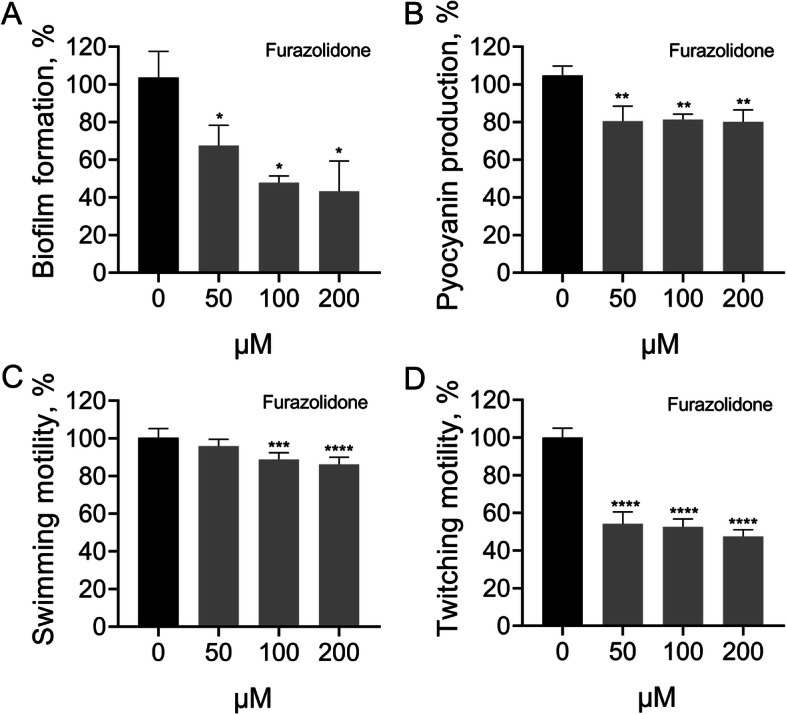
Inhibitory effect of furazolidone on the **A** biofilm formation, **B** pyocyanin production, **C** swimming motility and **D** twitching motility of *P. aeruginosa* PAO1. Data shown are the mean ± SD of three independent experiments, compare to the untreated control. One-way ANOVA, * *p* < 0.05, ** *p* < 0.01, *** *p* < 0.001, **** *p* < 0.0001

We found that furazolidone also showed a dose- and time-dependent growth inhibition on *P. aeruginosa* PAO1. However, the cell densities of *P. aeruginosa* PAO1 decreased slowly and remained relatively stable when the concentration of furazolidone reached 50 μM (Additional file 1 Supplementary Fig. S2 and S3). Considering the similar core structure of furazolidone and AHL signals, we then tested the possibility that furazolidone might negatively regulate the QS system of *P. aeruginosa* PAO1. Rapid population proliferation of *P. aeruginosa* using adenosine or skim milk as the sole carbon source requires the QS induced intracellular hydrolase or extracellular proteases, respectively [21]. Therefore, the growth status of *P. aeruginosa* under these conditions can be used to preliminarily evaluate the performance of QS system. As shown in Table 1, furazolidone significantly inhibited the growth of *P. aeruginosa* PAO1 on M9-adenosine plates, and had a dose-dependent inhibition effect on the production of extracellular proteases on M9-skim milk plates. Moreover, the production of biofilm and pyocyanin, and the swimming and twitching motilities of *P. aeruginosa* PAO1 could also be significantly inhibited by furazolidone (Fig. 3).

**Table 1:** Inhibitory effect of furazolidone on the growth of *P. aeruginosa* PAO1 on M9-adenosine and M9-skim milk plates

Furazolidone (μM)	M9-adenosine^a^	M9-skim milk (cm)^b^
0	+	1.72 ± 0.061
50	–	1.57 ± 0.050****
100	–	1.41 ± 0.074****
200	–	1.32 ± 0.050****

^a^“+”, Normal growth. “–”, Inhibited growth

^b^Data shown are the diameters of proteolytic ring (includes the diameters of colony), compare to the untreated control. Mean ± standard deviation (*n* = 9). One-way ANOVA, **** *p* < 0.0001

### Furazolidone inhibits the QS-regulation of *P. aeruginosa*

**Fig. 4:**
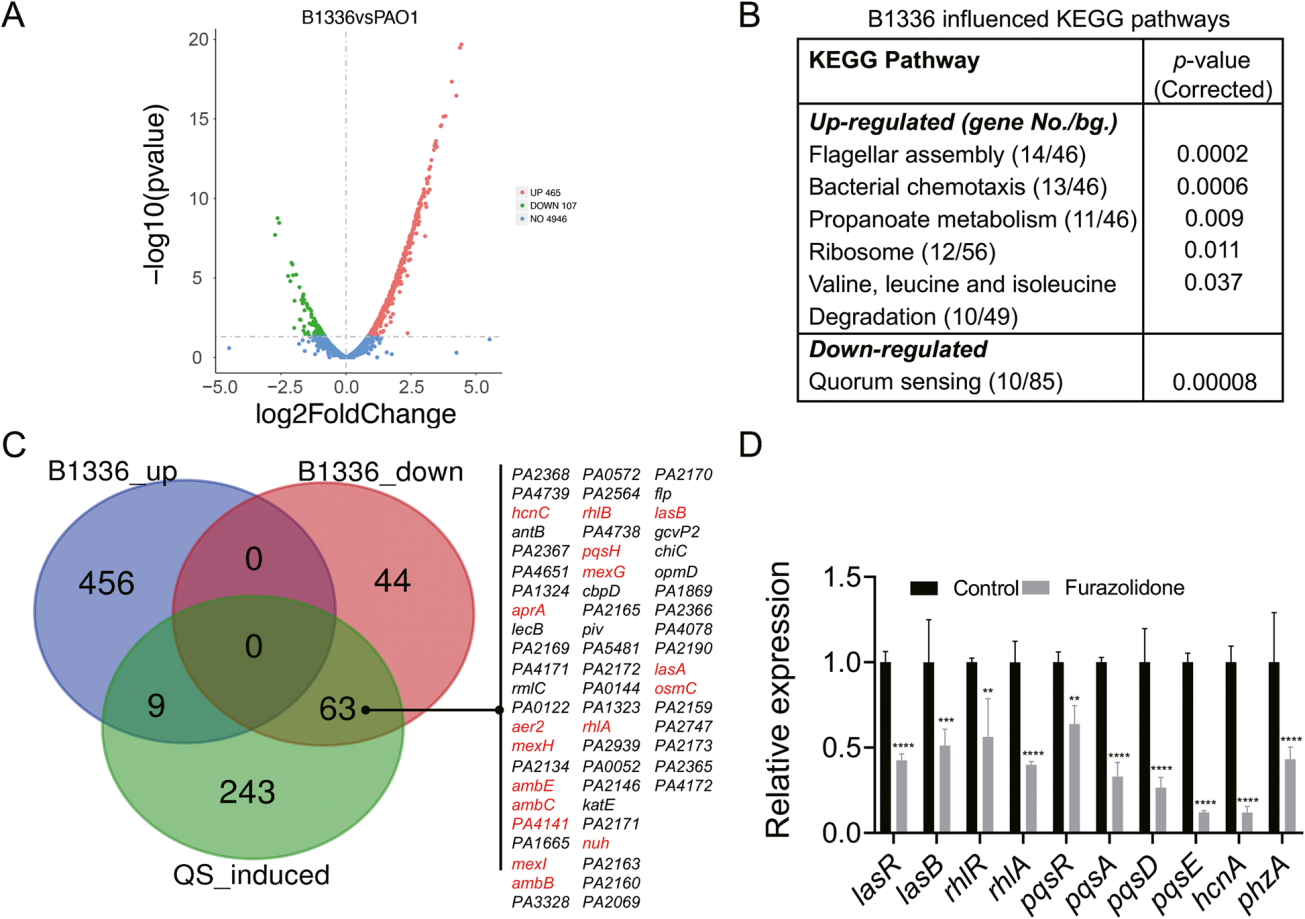
Effects of furazolidone on the global transcription of *P. aeruginosa* PAO1. **A** Volcano plotting of differentially expressed genes. B1336 indicates furazolidone treated PAO1. **B** Significantly influenced KEGG pathways of *P. aeruginosa* PAO1 by furazolidone. **C** The significantly differentially expressed genes of *P. aeruginosa* PAO1 were applied to the list of QS-induced genes published by Schuster et al. (2003). **D** Expression of typical QS-regulated genes of furazolidone-treated PAO1 determined by qPCR. Data shown are the mean ± SD of three independent experiments, compare to the untreated control. One-way ANOVA, ** *p* < 0.01, *** *p* < 0.001, **** *p* < 0.0001

To further investigate the effect of furazolidone on the QS-regulation of *P. aeruginosa*, RNA-sequencing was then used to profile the global transcription of furazolidone-treated *P. aeruginosa* PAO1. The results showed that compared to the control, 465 up-regulated genes and 107 down-regulated genes were identified in *P. aeruginosa* PAO1 cultured in LB broth supplemented with 200 μM of furazolidone (Fig. 4A and Additional file 2). Prediction of KEGG pathway revealed that the functions of flagellar assembly, bacterial chemotaxis, propanoate metabolism, ribosome, and degradation of valine, leucine and isoleucine were significantly enriched among the up-regulated genes (*p* < 0.05), while QS system was the sole significantly enriched KEGG term among the down-regulated genes (Fig. 4B).

We then explored the inhibitor effect of furazolidone on *P. aeruginosa* QS system in more detail by applying all the significantly changed genes to the list of QS-induced genes previously released by Schuster et al. [22]. The result that among the 315 QS-induced genes, 63 of them including the typical genes relating to the common QS-activated phenotypes, were screened from the 107 down-regulated genes (Fig. 4C). The result of quantitative PCR further confirmed that compared to the control, the expression levels of three key regulatory genes (*lasR*, *rhlR*, and *pqsR*) and their downstream functional genes (*lasB, rhlA, pqsA, pqsD, pqsE*, *hcnA*, and *phzA*) were all down-regulated by 1.5–8.2 fold in furazolidone-treated *P. aeruginosa* (Fig. 4D). Moreover, we also checked the effect of furazolidone on the expression levels of the main virulence factors of *T. pyogenes* TP13 and found that, the supplementation of furazolidone significantly decreased the expression of *plo*, *ploS*, *ploR*, *cbpA*, *fimA*, *nanH*, and *nanP* by 1.7–10.7 fold compared to that of untreated group (Additional file 1 Supplementary Fig. S4).

### Furazolidone protects *C. elegans* from *P. aeruginosa-T. pyogenes* co-infection

**Fig. 5:**
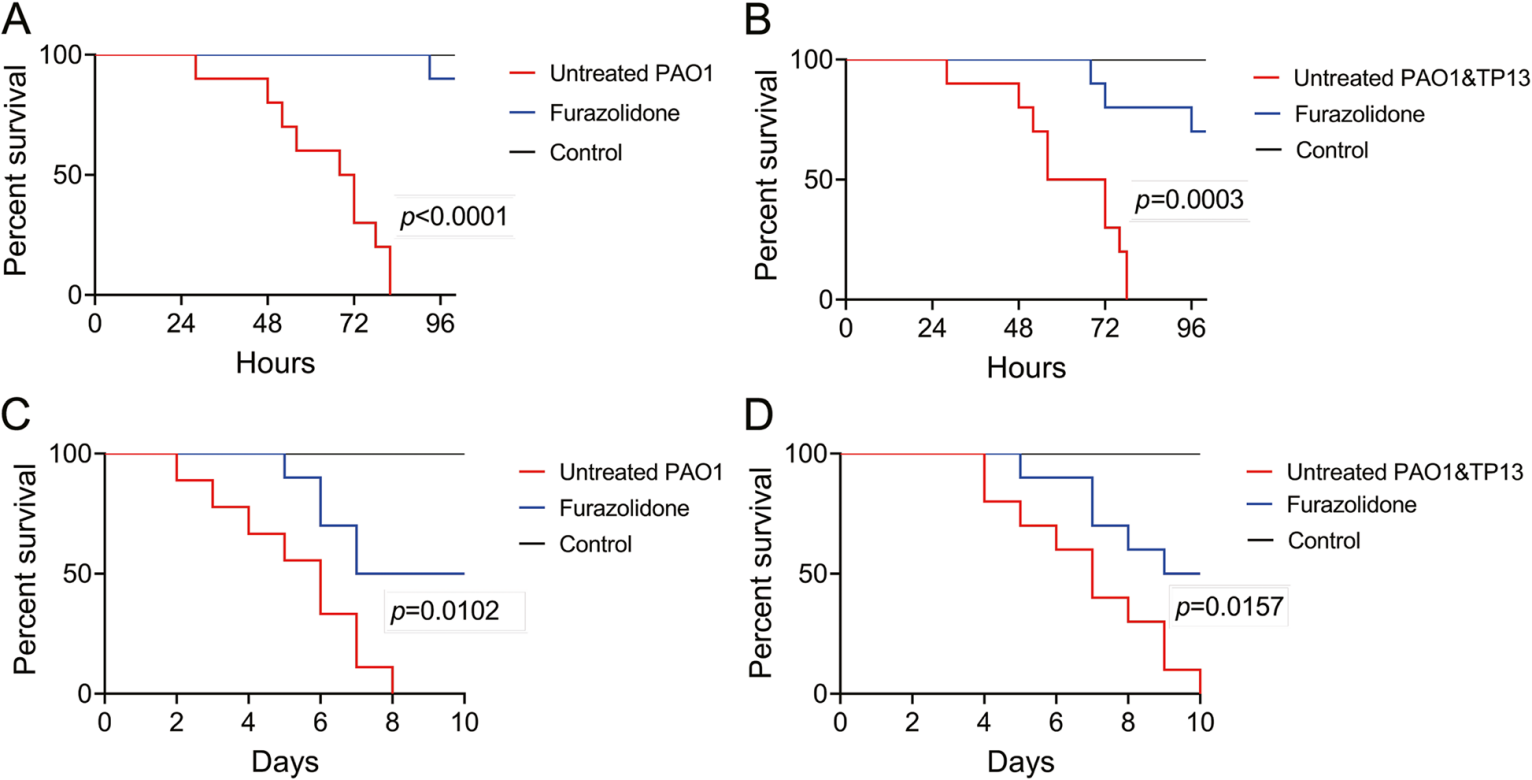
Furazolidone protects *Caenorhabditis elegans* from *P. aeruginosa-*infection and *T. pyogenes* + *P. aeruginosa* co-infection. Survival rate of *C. elegans* models challenged by (**A** and **C**) mono-cultured *P. aeruginosa* PAO1 or (**B** and **D**) 1:1 mixture of *T. pyogenes* TP13 and *P. aeruginosa* PAO1 in the (**A** and **B**) fast-killing assay or (**C** and **D**) slow-killing assay (10 *C. elegans* per group). Data shown are representative of six independent replicates, compare to the untreated group. The survival curves were compared by using Log-rank (Mantel-Cox) test

We then tested the in vivo protection activity of furazolidone against *P. aeruginosa* infection by using *C. elegans* as a model. In the fast-killing assay which mimics the acute infection condition, all the *C. elegans* were killed by mono-cultured *P. aeruginosa* PAO1 or co-cultured *P. aeruginosa* PAO1 and *T. pyogenes* TP13 (1:1) in 80 h in the untreated group (Fig. 5A, B), while furazolidone treatment significantly increased the survival rates of *C. elegans* under both infection conditions (*p* < 0.0001 and *p* = 0.0003, respectively). In the slow-killing assay which mimics the chronic infection condition, all the *C. elegans* were killed by mono-cultured *P. aeruginosa* PAO1 in 8 days on blank plates (Fig. 5C), and by co-cultured *P. aeruginosa* PAO1 and *T. pyogenes* TP13 (1:1) in 10 days (Fig. 5D). Expectedly, the presence of furazolidone significantly protected *C. elegans* from the challenges of pure *P. aeruginosa* PAO1 (*p* = 0.0102) and co-cultured *P. aeruginosa* PAO1 and *T. pyogenes* TP13 (*p* = 0.0157), albeit the protection effect was not comparable to that in fast-killing assay.

## Discussion

Purulent disease is a common disease greatly limits the population increase of forest musk deer and the development of musk-related industrials. The rapid emergence and spread of bacterial antibiotic resistance bring a huge challenge for the treatment of infectious diseases [23, 24]. Our prior work identifies that *T. pyogenes* is the causative agent of forest musk deer purulent disease, while *P. aeruginosa* dominates the development of respiratory suppurative lesions and leads the forest musk deer to an untimely death [2]. In the present study, we further report that furazolidone was capable of simultaneously inhibiting the growth and virulence of *T. pyogenes* and *P. aeruginosa*, and thus might be a promising candidate for the treatment of infections caused by the two species.

*T. pyogenes* is a Gram-positive bacterium with small genome size (≈ 2.3 Mbp) and commonly inhabits in the mucosal tissues of ruminant animals [6, 7, 10]. By contrast, the Gram-negative bacterium *P. aeruginosa* has a relatively large genome size (≈ 6.3 Mbp) and complex transcriptional network. These characters provide *P. aeruginosa* an innate advantage in colonizing different environments and resisting the clearance of antibiotics [14, 16, 25]. The present study confirmed the competitive advantage of *P. aeruginosa* over *T. pyogenes* by showing that, *P. aeruginosa* was always the dominant species in the co-culture with *T. pyogenes*, irrespective of their initial ratios (Fig. 2). This also provides an explanation for the replacement of dominant bacteria from *T. pyogenes* to *P. aeruginosa* during the development of suppurative lesions in forest musk deer lungs.

Reducing bacterial virulence by antagonizing the QS system, rather than killing bacterial cells or halting the growth of them, has been suggested to be an evolutionarily robust anti-infectious strategy [18, 26]. Previous studies have identified that the AHL signals of *P. aeruginosa* QS system could inhibit the growth and virulence of *T. pyogenes* in vitro and in vivo [4, 6, 19, 27]. In the present study, we first tested the antibacterial activities of 55 compounds on mono-cultured *T. pyogenes* and co-cultured *T. pyogenes* and *P. aeruginosa*. Furazolidone, which could profoundly inhibit the growth of *T. pyogenes* TP13 and slightly inhibit *P. aeruginosa* PAO1, was finally screened. Moreover, compared to the control groups, 200 μM of furazolidone was found to be capable of inhibiting the growth of *T. pyogenes* and *P. aeruginosa* in the mixed colonies started from any ratios, albeit *P. aeruginosa* was always the dominant species (Figs. 1, 2, and Additional file 1 Supplementary Fig. S3).

Furazolidone is a nitrofuran antibiotic that can be used to treat gastrointestinal diseases such as dysentery, enteritis, and gastric ulcer caused by bacteria and protozoa [28]. In this study, we further discovered that sub-inhibitory furazolidone could significantly reduce the production of QS-regulated virulence factors, biofilm formation, and cell motilities of *P. aeruginosa* PAO1 (Table 1 and Fig. 3). It has been reported that the commonly used clinical antibiotics azithromycin, ceftazidime, and ciprofloxacin could function as QS inhibitors of *P. aeruginosa* to improve the clinical outcome of patients [29–31]. Some natural products such as the extract of *Dalbergia Trichocarpa* bark, baicalin (an active natural compound extracted from the traditional Chinese medicinal *Scutellaria baicalensis*), and sodium ascorbate could also inhibit the QS-related virulence of *P. aeruginosa* [32–34]. Our transcriptional analysis further revealed that (Fig. 4), furazolidone could simultaneously inhibit the expression levels of all the three core regulatory genes and their downstream functional genes of *P. aeruginosa* PAO1. Additionally, the expression levels of the known virulence factor-encoding genes in *T. pyogenes* were also inhibited by furazolidone (Additional file 1 Supplementary Fig. S4). Therefore, the results indicated that furazolidone might be a good candidate to inhibit the virulence of *T. pyogenes* and *P. aeruginosa*.

On the other hand, it is noticed that furazolidone significantly up-regulated the expression of some genes relating to the flagellar assembly and chemotaxis of *P. aeruginosa* PAO1 as determined by RNA-sequencing (Fig. 4B). This is contradictory to the reduced swimming and twitching phenotypes of *P. aeruginosa* PAO1 by the presence of furazolidone (Fig. 4C, D). We reasoned that this might be due to the reduced the number of *P. aeruginosa* cells in the colony by sub-inhibitory furazolidone (Additional file 1 Supplementary Fig. S2), which could promote the expression of a partial of cell motility-related genes (14 out of 46) but limit the expansion of the whole population. Nevertheless, the result of *C. elegans* killing experiments demonstrated that furazolidone could efficiently protect *C. elegans* from *P. aeruginosa* infection and *T. pyogenes* + *P. aeruginosa* co-infection, especially in the fast-killing assay (Fig. 5). We failed to measure the protection of furazolidone on *C. elegans* against *T. pyogenes* challenge in this study due to the low virulence of *T. pyogenes* compared to the common bacterial pathogens. The *C. elegans* models grew well even when the inoculum dose of *T. pyogenes* was up to 1.0×10^9^ CFUs (Data not shown).

## Conclusion

Collectively, this study finds that furazolidone can simultaneously inhibit the growth and virulence of the two important bacterial pathogens of forest musk deer, *T. pyogenes* and *P. aeruginosa*. It is concluded that furazolidone can be considered as a promising candidate with antibacterial and anti-virulence capacities for the treatment of purulent disease of forest musk deer. The functional identification of furazolidone also provides a structural basis for development of novel anti-infectious drugs in the future.

## Methods

### Bacterial strains and media

*T. pyogenes* TP13 isolated from the lung pus of forest musk deer [6] and wild-type (WT) *P. aeruginosa* strain PAO1 were preserved in the lab and used elsewhere [17]. All the strains were routinely cultured in brain heart infusion with 5% fetal bovine serum (BHI-FBS) or in lysogeny broth (LB) from a single colony.

### Culture conditions

A total of 55 compounds (Additional file 1 Supplementary Table S1) with similar core structure to the Acyl-homoserine lactones (AHL) signals of *P. aeruginosa* QS system were selected and purchased from MedChemExpress (Shanghai, China). Overnight cultured *T. pyogenes* TP13 was diluted to optical density of 1.0 at wavelength of 600 nm (OD_600_ = 1.0) by sterile saline solution. Equal amount of *T. pyogenes* (10 μL) was inoculated in 200 μL of BHI-FBS medium containing different concentrations (0, 50, 100, and 200 μM) of compounds and cultured overnight at 37 °C. The cell densities of the culture were determined by measuring the OD_600_ and counting the colony forming units (CFUs) on BHI-FBS agar plates after appropriate dilution. Subsequently, *T. pyogenes* TP13 and *P. aeruginosa* PAO1 were mixed (1:9, 1:1, and 9:1) and co-cultured overnight on BHI-FBS agar containing 200 μM of the compounds with significant growth inhibition activities on *T. pyogenes*. The composition of *T. pyogenes* and *P. aeruginosa* in the co-culture were determined by counting the CFU of them on BHI-FBS agar plates after appropriate dilution, because the phenotypes of *T. pyogenes* and *P. aeruginosa* are significantly different and can be easily discriminated. Finally, the compounds that could inhibit the growth of *T. pyogenes* and *P. aeruginosa* were added (0, 50, 100, and 200 μM) to 200 μL of LB medium containing 10 μL of *P. aeruginosa* and cultured overnight at 37 °C. The cell densities were determined at OD_600_, and the colony forming units (CFUs) were counted on LB agar plates after appropriate dilution. All the experiments above were independently repeated for three times.

### Quorum-sensing inhibition assay

M9-adenisine (0.1%, wt/v) and M9-skimmed milk (0.5%, wt/v) agar medium were used to evaluate the inhibitory activity of compounds on *P. aeruginosa* QS regulation [21]. Overnight cultured *P. aeruginosa* PAO1 was adjusted to OD_600_ = 1.0 and inoculated (5 μL) on M9-adenisine agar and M9-milk agar containing different concentrations (0, 50, 100, and 200 μM) of compounds. The growth status of *P. aeruginosa* on M9-adenisine plates and the diameter of proteolytic circle formed on M9-milk plates were determined after 24 h. The experiments were independently repeated for nine times.

### Biofilm production assay

Equal amount (20 μl, OD_600_ = 1.0) of *P. aeruginosa* PAO1 was inoculated in glass tubes containing 2 mL of LB broth supplemented with different concentrations (0, 50, 100, and 200 μM) of compounds, and overnight cultured at 37 °C with shaking (220 rpm). The cell density was measured at OD_600_. After the culture solution and unadhered biofilm were gently removed, the adhered biofilm on the tube wall was stained with crystal violet (0.1%) for 30 min and washed with PBS buffer for three times. Subsequently, the stained biofilm was dissolved by 95% of ethanol solution and quantified at OD_595_. The experiments were independently repeated for three times.

### Pyocyanin production assay

Equal amount (20 μl, OD_600_ = 1.0) of *P. aeruginosa* PAO1 was inoculated in glass tubes containing 2 mL of LB broth supplemented with different concentrations (0, 50, 100, and 200 μM) of compounds, and overnight cultured at 37 °C with shaking (220 rpm). After the cell density was equalized with fresh LB broth, 200 μL of bacterial solution was taken out to extract the pyocyanin by chloroform and 0.2 N HCl and measured at OD_520_ as described by Essar [35]. The experiments were independently repeated for three times.

### Motility assay

For the swimming motility assay, 5 μl (OD_600_ = 1.0) of *P. aeruginosa* PAO1 was inoculated on the surface of LB plates containing 0.5% of agar supplemented with different concentrations (0, 50, 100, and 200 μM) of compounds and cultured at 37 °C for 24 h. For the twitching motility assay, 2 μl (OD_600_ = 1.0) of *P. aeruginosa* PAO1 was stabbed into the bottom of LB plates containing 1.0% of agar supplemented with different concentrations (0, 50, 100, and 200 μM) of compounds and cultured at 37 °C for 24 h. The motilities of *P. aeruginosa* PAO1 were determined by measure the diameters of colony on the surface (swimming motility) or the thin film region on the bottom (twitching motility). All the experiments were independently repeated for six times.

### Transcriptomic analysis

Bacterial cells of furazolidone-treated (200 μM) and -untreated *P. aeruginosa* PAO1 were harvested for total RNA isolation using TRIzol reagents (Invitrogen), respectively. RNAs samples were conducted for library construction and RNA-sequencing (RNA-seq) by Novogene Bioinformatics Technology Company using prokaryotic strand-specific Illumina-based RNA-Seq technology (HiseqTM2500 platform). The obtained clean reads were mapped to the reference genome of PAO1 (NCBI accession number: AE004091) by the software Tophat2 [36]. SOAP2 program [37] and Cufflinks [38] were used to calculate the expected fragments per kilobase of transcript per million fragments (FPKM) sequenced, and the differentially expressed transcripts were presented and analyzed by EdgeR [39]. Differentially expressed gene with false discovery rate *p* < 0.05 was thought to be significantly different. The significantly differently expressed genes were mapped to the list of QS-activated genes reported by Schuster et al. [22] using Venn Diagrams (http://bioinformatics.psb.ugent.be/webtools/Venn/).

### Quantitative PCR

Total RNAs of furazolidone-treated and -untreated *P. aeruginosa* PAO1 were isolated by using TRIzol reagents, and the cDNA was synthesized by reverse transcription using a high-capacity cDNA Reverse Transcriptase kit Specific with gDNA removal (Takara). Quantitative PCR was performed by using an iTaq™ universal SYBR® Green Supermix (Bio-Rad) and the CFX Connect Real-Time PCR Detection System to validate the expression of typical QS-activated genes including *lasR*, *rhlR*, *pqsR*, *lasB*, *rhlA*, *pqsA*, *pqsD*, *pqsE*, *hcnA*, and *phzA* (Additional file 1 Supplementary Table S2). Gene expression was calculated by the 2^−ΔΔCT^ method using 16S rRNA as reference.

### *Caenorhabditis elegans* assay

For the fast-killing assay, 20 μl (OD_600_ = 1.0) of *P. aeruginosa* PAO1 and mixture of *T. pyogenes* TP13 and *P. aeruginosa* PAO1 (1:1) were spread on peptone-glucose-sorbitol (PGS) agar media with and without small molecule drug respectively, and cultured overnight at 37 °C. The naturally cooled plates were seeded with 10 newly cultured adult *C. elegans* (L4 stage) and further incubated at 25 °C for 96 h. For the slow-killing assay, to prevent *C. elegans* from laying eggs, 40 μL of 5-fluoro-2′-deoxyuridine solution (40 μg/mL) was evenly coated on the surface of nematode growth medium (NGM). Subsequently, 20 μl (OD_600_ = 1.0) of *P. aeruginosa* PAO1 and mixture of *T. pyogenes* TP13 and *P. aeruginosa* PAO1 (1:1) were spread on NGM plates with and without small molecule drug and cultured overnight at 37 °C. The naturally cooled plates were seeded with 10 newly cultured adult *C. elegans* and further incubated at 25 °C for 10 days. The survival status of *C. elegans* in each experiment were observed and recorded. Growth of *C. elegans* on PGS agar plates or NGM plates feed with uracil auxotrophy *Escherichia coli* OP50 were set as controls.

### Statistical analyses

Data analysis and statistical tests were performed by using Graphpad Prism version 9.0 (San Diego, CA, USA). Mean values of standard deviation were compared by using two-tailed unpaired t-test or One-way ANOVA. The survival curves of *C. elegans* were compared by using Log- rank (Mantel-Cox) test.

## Supplementary Information

**Additional file 1.**

**Additional file 2.**

## Data Availability

The data shown in this paper are available within the article and supplementary materials. The raw data of RNA- sequencing are available from the NCBI database under accession number PRJNA723215 (SRR14368535 and SRR14368537) https://www.ncbi.nlm.nih.gov/sra/?term=PRJNA723215.

## Abbreviations

AHL: Acyl-homoserine lactones
BHI: Brain heart infusion
CFUs: Colony forming units
FBS: Fetal bovine serum
FPKM: Fragments per kilobase of transcript per million fragments
LB: Lysogeny broth
NGM: Nematode growth medium
PCR: Polymerase chain reaction
PGS: Peptone-glucose-sorbitol
QS: Quorum-sensing
WT: Wild-type
