# Supplementary material for: Antibacterial and anti-virulence effects of furazolidone on Trueperella pyogenes and Pseudomonas aeruginosa
Source: BMC Vet Res. 2022 Mar 24;18:114. doi: 10.1186/s12917-022-03216-5 (PMC8943969; doi:10.1186/s12917-022-03216-5)
Supplement: Supplementary file 1 — Additional file 1. [file 12917_2022_3216_MOESM1_ESM.docx]

**Antibacterial and anti-virulence effects of furazolidone on *Trueperella pyogenes* and *Pseudomonas aeruginosa***

Qin Chen^a^, Kelei Zhao^b,^*, Heyue Li^a^, Kanghua Liu^a^, Jing Li^b^, Yiwen Chu^b^, Balakrishnan Prithiviraj^c^, Bisong Yue^a^, Xiuyue Zhang^a,^*

^a^ Key Laboratory of Bio-resources and Eco-environment, Ministry of Education, College of Life Sciences, Sichuan University, Chengdu, 610064, China.

^b^ Antibiotics Research and Re-evaluation Key Laboratory of Sichuan Province, College of Pharmacy, Sichuan Industrial Institute of Antibiotics, Chengdu University, Chengdu 610052, China.

^c^ Marine Bio-products Research Laboratory, Department of Plant, Food and Environmental Sciences, Dalhousie University, Truro, NS, Canada.

* Corresponding author at: Antibiotics Research and Re-evaluation Key Laboratory of Sichuan Province, College of Pharmacy, Sichuan Industrial Institute of Antibiotics, Chengdu University, No. 168 Huaguan Road, 610052, Chengdu, Sichuan, PR China; Key Laboratory of Bio-resources and Eco-environment, Ministry of Education, College of Life Sciences, Sichuan University, No. 24, South Section 1, Yihuan Road, 610064, Chengdu, Sichuan, PR China.

E-mail addresses: zhaokelei@cdu.edu.cn (K. Zhao), zhangxiuyue@scu.edu.cn (X. Zhang).

## Supplementary Figures


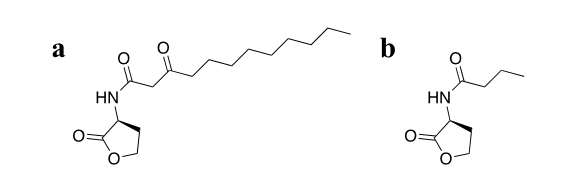
 **Supplementary Fig. S1.** Chemical structures of the signal molecules of *Pseudomonas aeruginosa* quorum-sensing system. **a**, N-(3-oxo-dodecanoyl)-L-homoserine lactone (OdDHL). **b**, N-butanoyl-L-homoserine lactone (BHL)

**
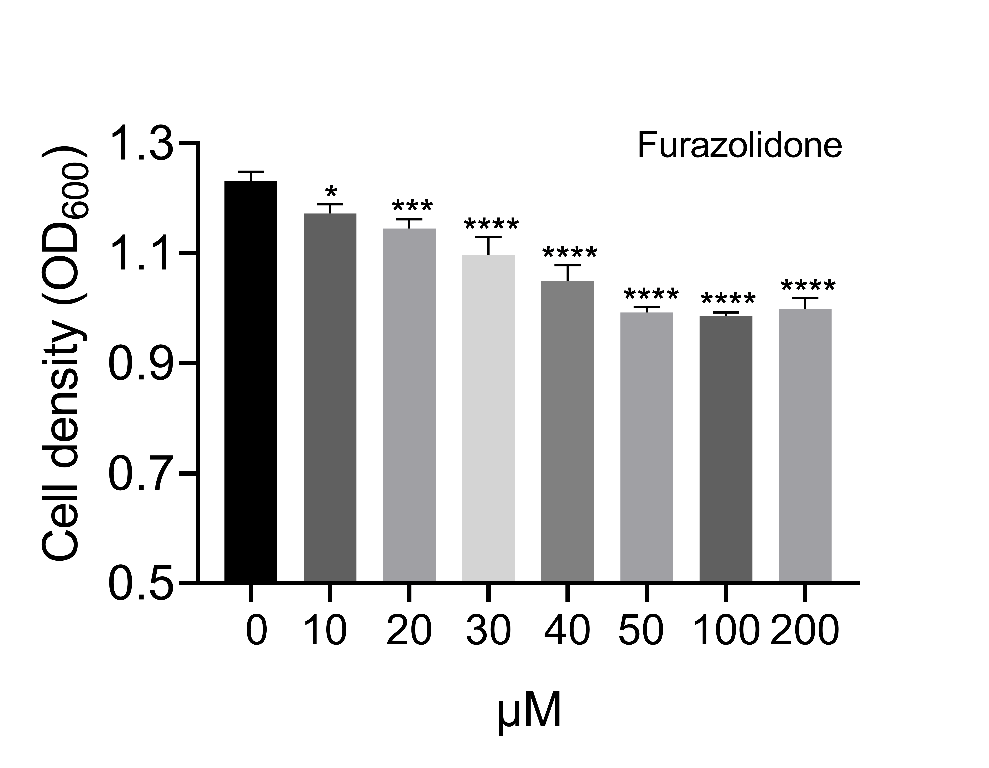
**

**Supplementary Fig. S2.** Effect of furazolidone on the growth of *P. aeruginosa* PAO1 cultured in lysogeny broth (LB) broth. Date shown the mean ± standard deviation (SD) of three independent experiments, compare to the untreated control. One-way ANOVA, * *p* < 0.05, *** *p* < 0.001, **** *p* < 0.0001.

A


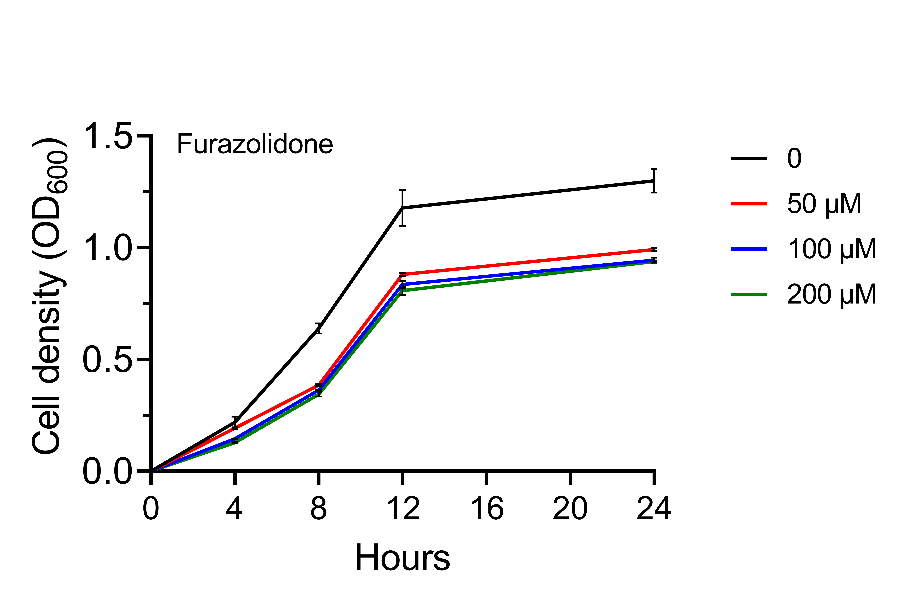


B


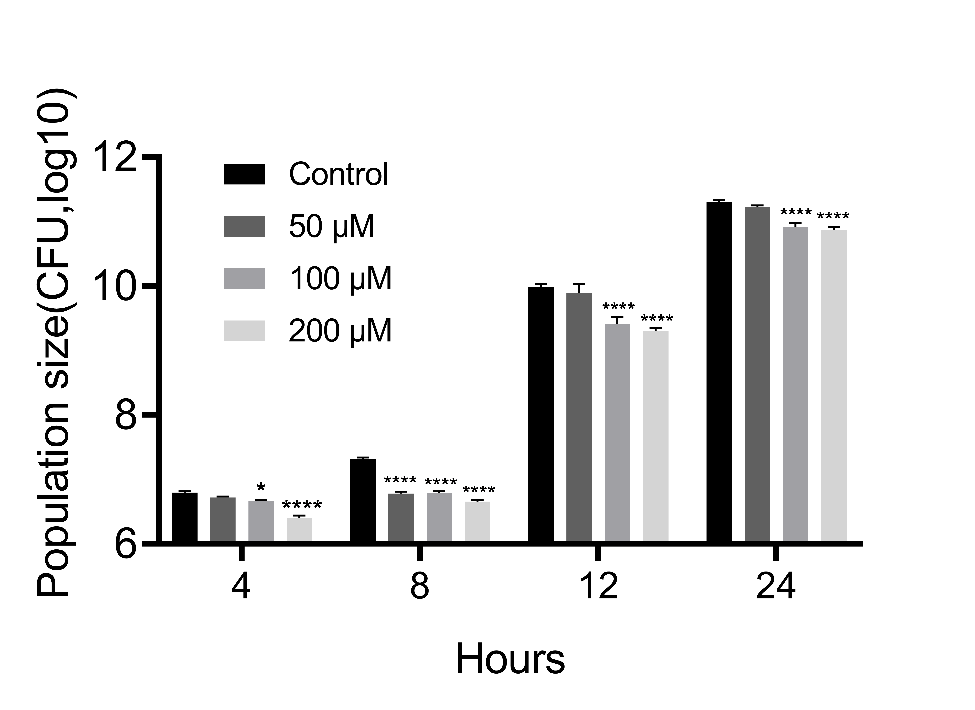


**Supplementary Fig. S3.** Effect of furazolidone on the growth of *P. aeruginosa* PAO1 cultured in lysogeny broth (LB) broth at different time points. A. Cell density (OD_600_) B. Population size (CFU, log10). Date shown the mean ± SD of three independent experiments, compare to the untreated control. One-way ANOVA, * *p* < 0.05, *** *p* < 0.001, **** *p* < 0.0001.


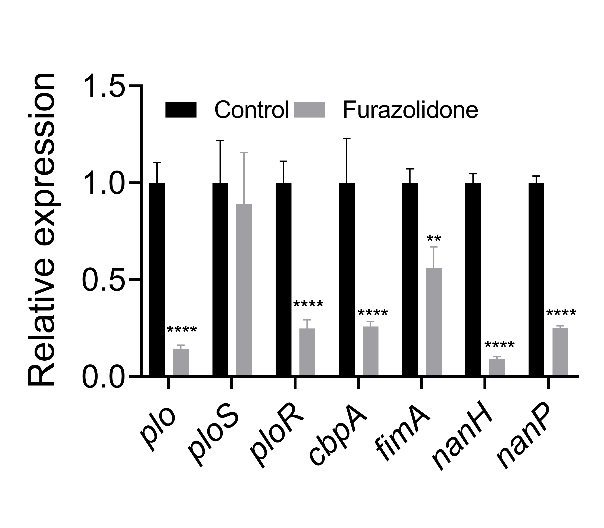


**Supplementary Fig. S4.** Effect of furazolidone on the expression of virulence-related genes of *Trueperella pyogenes* TP13 determined by qPCR. Date shown the mean ± SD of three independent experiments, compare to the untreated control. Unpaired t-test: ** *p* < 0.01, **** *p* < 0.0001.

## Supplementary Tables

**Supplementary Table S1.** Effect of 55 compounds on the growth of *T. pyogenes* TP13*.*

| Compounds | Effect on growth^a^ |
| --- | --- |
| 2,5-Dihydroxyphenylacetic acid lactone | No change |
| 2,5-Furandicarboxylic acid | No change |
| 5-Hydroxymethylfurfural | No change |
| Sesamol | No change |
| 2-Benzoxazolinone | No change |
| 3-Indoleacetic acid | No change |
| Carbendazim | No change |
| L-5-Hydroxytryptophan | No change |
| Fenbendazole | No change |
| 5-Hydroxymethyl-2-furancarboxylic acid | No change |
| L-Pyroglutamic acid | No change |
| Sulfathiazole sodium | Decrease |
| Succinylsulfathiazole | No change |
| Tazobactam | Decrease |
| 5-Methyluridine | No change |
| Acadesine phosphate | No change |
| 2'-Deoxyguanosine | No change |
| Uracil 1-β-D-arabinofuranoside | No change |
| Kinetin (6-Furfuryladenine) | No change |
| Cotinine | No change |
| Creatinine | No change |
| Arctigenin | No change |
| Methoxsalen (8-Methoxypsoralen) | No change |
| Harmine | Decrease |
| Nifuratel (Methylmercadone) | Decrease |
| Oxaceprol (N-Acetyl-L-hydroxyproline) | No change |
| Pilocarpine Hydrochloride | No change |
| Metronidazole | No change |
| Nifursol | Increase |
| Nithiamide | No change |
| **Furazolidone** | **Decrease** |
| **Nitrofurantoin** | **Decrease** |
| **Nitrofurazone** | **Decrease** |
| Ribavirin | No change |
| Dimetridazole | No change |
| Dinotefuran | Increase |
| **Ronidazole** | **Decrease** |
| Tizoxanide | No change |
| Bisantrene | Increase |
| Andrographolide | No change |
| Ursodiol | No change |
| Bilobalide | Increase |
| 5-Amino-3H-imidazole-4-Carboxamide | Increase |
| Allantoin | No change |
| Methyl 2-furoate | No change |
| 5-Methylfurfural | Increase |
| Aminothiazole | Increase |
| Chloramphenicol | Decrease |
| 11β-Hydroxyandrosterone | Increase |
| Urocanic acid | No change |
| 3-Formyl-2-phenylindole | No change |
| DL-3-Phenyllactic acid | No change |
| D-Cycloserine | No change |
| Timepidium bromide | No change |
| 14-Deoxy-11,12-didehydroandrographolide | No change |

All of *T. pyogenes* were cultured in BHI+5% FBS broth and independently repeated for three times.

^a^ Compared with *T. pyogenes* treated by DMSO or water, un-paired t test, *p* < 0.05. Bold color indicates *p* < 0.01.

**Supplementary Table S2**. Primers used in this study

| Gene | Sequence (5’-3’) |
| --- | --- |
| *lasR*rtF | CTTCATCGTCGGCAACTAC |
| *lasR*rtR | GTCTGGTAGATGGACGGTTC |
| *lasB*rtF | ATCGGCTACGACATCAAGAAGG |
| *lasB*rtR | CCGCTGTTGTAGTTGCTGGTG |
| *rhlR*rtF | GCTCCTCGGAAATGGTGGT |
| *rhlR*rtR | GGAAAGCACGCTGAGCAAAT |
| *rhlA*rtF | ACTGAACCAGGCGATGCTC |
| *rhlA*rtR | GCTCCAGGCAAGCCAAGTA |
| *pqsR*rtF | CACTGGTTGAAGCGGGAGA |
| *pqsR*rtR | TCGTTCTGCGATACGGTGAG |
| *pqsA*rtF | GCTGAGCGGTCCTTTGGC |
| *pqsA*rtR | TGGAACCCGAGGTGTATTGC |
| *pqsD*rtF | GCTGTACGGCTTGCAGATGG |
| *pqsD*rtR | CAGGTCCAGCAGTCCGTCTT |
| *pqsErt*F | ATGTTGAGGCTTTCGGCT |
| *pqsErt*R | GCAGTGGTCGTAGTGCTTGTG |
| *hcnArt*F | GCAGACATGACCATCCACCTC |
| *hcnArt*R | CGGTTGCTTTCGGTTTCCA |
| *phzA1rt*F | GCAACTGGACCACGGAAAG |
| *phzA1rt*R | GCACGCAGTTTCTGTATCGG |
| *16Srt*F | TCGCATCCTGTTGTCCTCCA |
| *16Srt*R | TTAGCCAGGGTCAGCGTCA |
